# Identification of a Novel Antimicrobial Peptide From the Ancient Marine Arthropod Chinese Horseshoe Crab, *Tachypleus tridentatus*

**DOI:** 10.3389/fimmu.2022.794779

**Published:** 2022-03-23

**Authors:** Wei-Feng Wang, Xiao-Yong Xie, Yan Huang, Yin-Kang Li, Hong Liu, Xiu-Li Chen, Huan-Ling Wang

**Affiliations:** ^1^ Key Lab of Freshwater Animal Breeding, Key Laboratory of Agricultural Animal Genetics, Breeding and Reproduction, Ministry of Education, College of Fisheries, Huazhong Agricultural University, Wuhan, China; ^2^ Key Laboratory of South China Sea Fishery Resources Exploitation & Utilization, Ministry of Agriculture, South China Sea Fisheries Research Institute, Chinese Academy of Fishery Sciences, Guangzhou, China; ^3^ Guangxi Key Laboratory of Aquatic Genetic Breeding and Healthy Aquaculture, Guangxi Academy of Fishery Sciences, Nanning, China

**Keywords:** *Tachypleus tridentatus*, antimicrobial peptide, LPS, cysteine-stabilized motif, broad-spectrum

## Abstract

Humoral immunity is the first line of defense in the invertebrate immune system, and antimicrobial peptides play an important role in this biological process. A novel antimicrobial peptide, termed Tatritin, was identified and characterized in hemolymph of Chinese horseshoe crab, *Tachypleus tridentatus*, infected with Gram-negative bacteria *via* transcriptome analysis. Tatritin was significantly induced by bacterial infection in hemolymph and gill. The preprotein of Tatritin consists of a signal peptide (21 aa) and a mature peptide (47 aa) enriched by cysteine. The putative mature peptide was 5.6 kDa with a theoretical isoelectric point (pI) of 9.99 and showed a *α*-helix structure in the N-terminal and an anti-parallel *β*-sheet structure in the cysteine-stabilized C-terminal region. The chemically synthesized peptide of Tatritin exhibited a broad spectrum of antimicrobial activity against Gram-negative and Gram-positive bacteria and fungi. Furthermore, Tatritin may recognize and inhibit pathogenic microorganisms by directly binding to LPS, DNA, and chitin. In addition, administration of Tatritin reduced the mortality of zebrafish after bacterial infection. Due to its broad-spectrum antimicrobial activity *in vivo* and *in vitro* and the sensitivity to drug-resistant bacterial strains, Tatritin peptide can be used as a new type of drug for infection treatment or as an immune enhancer in animals.

## Introduction

Chinese horseshoe crab (*Tachypleus tridentatus*) is an ancient marine arthropod. Although the horseshoe crab has an evolutionary history of more than 450 million years, its shape has hardly changed compared with its ancient ancestors in the past 200 million years (1–3). The current view holds that marine invertebrates lack an acquired immunity, and the defense system against infectious agents is mediated by innate immunity, including humoral and cellular responses (4). Humoral immunity in horseshoe crab is characterized by antimicrobial substances present in the hemolymph plasma and hemocytes, along with reactions such as hemolymph coagulation or melanization (5, 6). Horseshoe crab hemocytes are highly sensitive to lipopolysaccharides (LPS), peptidoglycan, and *β*-1,3-glucans, which are major outer membrane components of Gram-negative and -positive bacteria and fungi, respectively (7). The defense molecules, such as antimicrobial peptides (AMPs), lectins, serine protease zymogens, and coagulation factors, stored in granules of hemocytes in horseshoe crab are secreted by exocytosis after stimulation with pathogenic microbes (5, 8, 9).

AMPs are important components of the non-specific host defense or innate immune system in a variety of organisms ranging from microbes to plants and animals including mollusks, arthropods, amphibians, and mammals (10, 11). Generally, the AMPs are small, cationic, cys-stabilized, and amphipathic molecules. Over an evolutionary time span, these peptides have retained potency against pathogen invasion, in the face of highly mutable target microorganisms. These cationic AMPs can change the electrochemical potential of bacteria *via* binding and interacting with negatively charged bacterial cell membranes, which induces cell membrane damage and allows for penetration of larger molecules, such as proteins, destroying cell morphology and ultimately leading to cell death (12). So far, four membrane-targeting mechanisms, based on the carpet model, the barrel stave model, the toroidal pore model, and the agglutination model, have been proposed in previous studies for different AMPs (13, 14). Therein, in the carpet model, the barrel stave model, and the toroidal pore model, AMPs act on the cell membrane *via* their special structure (such as cationic, amphiphilic, and hairpin structure) and arrangement, thereby causing membrane damage. The agglutination model differs from other models in that its action mechanism does not involve membrane damage. The anti-lipopolysaccharide factor (ALF) isolated from horseshoe crab promotes the pathogens to be phagocytosed by the host cells and the release of toxic substances to be reduced *via* the aggregation of multiple bacterial cells (15, 16). The non-specific and complex action modes make AMPs less likely to cause bacterial resistance than antibiotics (17). Recently, many studies have shown that AMPs can directly kill pathogens by binding to non-membrane targets of cell walls or intracellular components to inhibit the biologic synthesis of intracellular proteins, nucleic acids, lipoteichoic acid, peptidoglycan, and other key biomolecules (18). In addition, AMPs can also exert their effects *via* immune regulation mechanisms to inhibit pathogens. Some AMPs are involved in the differentiation of relevant immune cells and activation of immune cells (macrophages, monocytes, dendritic cells, and T cells) (19–21). As known, AMPs have broad-spectrum antimicrobial activities against bacteria, fungi, and virus, and even some AMPs have anticancer activity (2, 4, 12). As more antibiotics are rendered ineffective by drug-resistant bacteria, focus must be shifted toward alternative therapies for treating infections. The abundance of AMPs from different organisms may be effective alternative drugs against rapidly mutating and evolving pathogenic microorganisms (22, 23).

In previous studies, the AMPs, including Tachyplesins, big defensin, Tachycitin, and Tachystatins in Chinese horseshoe crab, have been functionally and structurally characterized (24–27). Tachyplesins (Tachyplesins I and II) are a family of cationic peptides composed of 17 amino acids and localized in small granules of hemocytes. Tachyplesin has an amphiphilic antiparallel *β*-sheet structure (positive charges and hydrophobic amino acids are distributed in two sides of the *β*-sheet), which seems important for antimicrobial activities (5, 28). Tachyplesin has LPS-binding activity and strong antimicrobial activity against Gram-negative and -positive bacteria and fungi (27). The big defensin is a mature peptide consisting of 79 amino acids, with a hydrophobic extension at the NH_2_ terminal and a cationic defensin region at the COOH terminal. Similar to Tachyplesins, it inhibits the growth of not only Gram-positive and -negative bacteria but also fungi. Furthermore, the NH_2_-terminal hydrophobic extension region shows growth inhibitory activity against Gram-positive bacteria and the COOH-terminal cationic defensin portion against Gram-negative bacteria (24). The cys-stabilized mature Tachycitin consisted of 73 amino acid residues containing five disulfide bonds. Although Tachycitin has inhibitory activity against Gram-positive and -negative bacteria and fungi, its antibacterial activity is relatively weak compared with big defensin (25). In fact, Tachycitin can enhance the antibacterial ability of big defensin through a synergistic effect. Tachystatins, including Tachystatin A, Tachystatin B, and Tachystatin C, are a family of antimicrobial peptides whose cys-stabilized structure is similar to some insecticidal neurotoxins of spider venom (26). Like the previously reported AMPs in Chinese horseshoe crab, Tachystatins also have a broad-spectrum antimicrobial effect and a strong growth inhibitory activity against Gram-negative and -positive bacteria and fungi. In addition, Tachystatins may change the cell morphology of fungi by binding chitin in the cell wall to inhibit cell growth. Although the cysteine structure of the three Tachystatin peptides is similar, only Tachystatin C exhibits hemolytic activity.

In the present study, to our knowledge, a new potential AMP gene, termed *Tatritin*, was identified and characterized in Chinese horseshoe crab hemolymph *via* transcriptome analysis postinfection with Gram-negative bacteria (29). Although Tatritin did not show similarity to reported sequences, it was characterized as a new AMP that is important for antibacterial immunity of Chinese horseshoe crab. Additionally, the Tatritin peptide has a broad antimicrobial spectrum *in vivo* and *in vitro* and can be used as a new type of drug for infection treatment or as an immune enhancer in animals.

## Materials and Methods

### Sample Collection

Artificially raised Chinese horseshoe crabs (120 ± 20 g) were obtained from an aquaculture research base of South China Sea Fisheries Research Institute, Chinese Academy of Fishery Sciences (Zhanjiang, China), maintained and acclimated to the laboratory conditions as previously described (29).

### Gene Cloning and Sequence Analysis

Chinese horseshoe crabs were euthanized, and total RNA was extracted from seven tissues, including intestine (midgut), muscle (the base of telson), gill, stomach (proventriculus), heart, hemolymph and hepatopancreas using TRIzol^®^ Reagent (Ambion, Life Technologies, Carlsbad, CA, USA) according to the manufacturer’s instructions. Quality and quantity of the extracted RNA were assessed by 1% agarose gel electrophoresis and using a NanoDrop 2000 spectrometer (Thermo Fisher Scientific, Waltham, MA, USA) with the threshold for A260/A280 > 1.8. First-strand cDNA was synthesized from the total RNA using a reverse transcription system (Promega, Madison, WI, USA) with oligo(dT)_18_ and pd(N)_6_ (Takara, Mountain View, CA, USA) primers and stored at -20°C.

According to our previous study (29), a putative antimicrobial peptide gene (termed *Tatritin* in this study) was identified in RNA-seq datasets (NCBI accession number SRP267502). The open reading frame (ORF) of *Tatritin* was amplified by PCR based on the transcript sequence. In addition, the primers for cloning the 3′ end of *Tatritin* cDNA were designed according to the described method (30).

PCR products were cloned into the pGEM T Easy vector (Promega) and sequenced. The ORF and signal peptide were predicted with the DNAStar_LaserGene 7.1 software (https://www.dnastar.com/) and SignalP online software (http://www.cbs.dtu.dk/services/SignalP/), respectively. Multiple-sequence alignment was generated using ClustalW2 (http://www.ebi.ac.uk/).

To analyze the three-dimension structure of Tatritin, the Tatritin peptide was modeled by the *ab initio* method using Rosetta 3.5 software (31) and dDFIRE (32) as previously described (33). In addition, the peptide was visualized using the Chimera 1.1 software (34), where views of surface of the Coulombic electrostatic potential and amino acid hydrophobicity were generated.

### Expression Patterns of the *Tatritin* Gene

Seven tissues from Chinese horseshoe crabs were used for expression pattern analysis of *Tatritin via* quantitative real-time PCR (qRT-PCR). Meanwhile, the relative expression levels of *Tatritin* in Chinese horseshoe crabs infected with *Vibrio parahaemolyticus* in different time points (0, 12, 24, 36, and 72 h) postinfection were determined by qRT-PCR. The challenge process was described in the previous study (29). qRT-PCR was conducted using a CFX Connect™ Real-Time PCR detection system (Bio-Rad Laboratories Inc., Hercules, CA, USA) as previously described (29). Expression levels of the *Tatritin* gene were calculated by 2^-△△Ct^ (35), and *gapdh* was selected as the reference gene (36).

### Peptide Synthesis and FITC Labeling

Tatritin peptide was synthesized and analyzed by HPLC and MALDI-TOF mass spectroscopy to confirm the purity with >95% in KE Biochem (Shanghai, China). The Tatritin peptide was labeled by FITC as previously described (37). Briefly, FITC was freshly dissolved in dimethyl sulfoxide (DMSO, Sigma, St. Louis, MO, USA) then added to the peptide in 100 mM sodium carbonate buffer (pH 9.3) to a final concentration of 1.96 mg/ml. The solution was incubated in the dark for 12 h at 4°C, and then NH_4_Cl (50 mM) was added to inactivate the residual FITC in the dark for 2 h at 4°C. The FITC-labeled peptide was purified by using a Focudex G-25 Medium (Huiyan Bio, Hainan, China) according to the manufacturer’s protocol. Then, the concentration of the FITC-labeled peptide was measured by the BCA method using a protein concentration determination kit (Biosharp, Hefei, China).

### Expression of Tatritin and Polyclonal Antibody Production

Tatritin was amplified and ligated to the pGEX-4T-1 expression vector, and the recombinant protein with a GST tag in the N-terminal was expressed in *E. coli* TSBL21 (DE3). Then, a Glutathione Sepharose 4 Fast Flow resin (Huiyan Bio, Wuhan, China) was used to clean and recover the recombinant protein in the PD-10 column according to the manual. Tris-SDS-PAGE (12%) was performed to assess the purity of recombinant protein.

The production and purification of polyclonal antibodies were performed by Dia-An Biotechnology Co., Ltd. (Wuhan, China). Briefly, the recombinant protein of GST-Tatritin was used to raise polyclonal antibodies in rabbits, and a specific anti-Tatritin antibody was harvested using the affinity column. Western blot was conducted to test the reactivity of the antibody with Tatritin using the other recombinant protein His-Tatritin expressed by *E. coli* TSBL21 using a pET-32a vector (data not shown).

### Immunofluorescence Localization of Tatritin in Muscle and Gill Tissues

To analyze the localization of Tatritin in different tissues of Chinese horseshoe crab, immunofluorescence analysis was performed on paraffin sections of muscle and gill tissues. Briefly, tissue sections were incubated with rabbit anti-Tatritin antibody (0.6 μg/ml), followed by CY3-conjugated goat anti-rabbit IgG antibody (2.5 μg/ml, Servicebio, Wuhan, China). Rabbit anti-GST antibody (0.6 μg/ml) was used as the negative control. After staining with DAPI (Servicebio, China), the images were acquired using a fluorescence microscope (Leica, Wetzlar, Germany).

### Antimicrobial Activity

Six bacterial strains, including *Aeromonas hydrophila*, *Escherichia coli* (ESBLs-EC, ATCC35218), *Klebsiella pneumoniae* (CMCC(B)46117), *Staphylococcus aureus* (ATCC6538), *Streptococcus agalactiae* (ATCC13813), and *S. aureus* (MRSA, ATCC43300), and one species of fungi, *Candida albicans* (ATCC10231), were used to test the antimicrobial activities of Tatritin. Antibacterial activity assay was performed by the 2-fold microtiter broth dilution method as previously described by Zhang et al. (38). Briefly, Tatritin peptides with the final concentrations of 1, 2, 4, 8, 16, 32, and 64 µM were added to a 96-well microtiter plate (Corning, Tewksbury, MA, USA) with 20 µl bacterial cells (2–5 × 10^5^ CFU/ml in LB broth). The wells without peptide were used as controls. Three replicates were set in all peptide concentrations. Initial OD_595_ was measured using an Infinite F200 microplate reader (Tecan, Grödig, Austria). Then these plates were incubated at 28°C for 12 h. OD_595_ was measured and corrected by initial OD_595_ values. Bacterial growth rates were calculated as the bacterial densities in the presence of peptides to the bacterial densities of controls. To test the inhibitory activity of Tatritin against fungi, *C. albicans* was used for antimicrobial assay. Briefly, approximately 2 × 10^5^ cells of *C. albicans* were mixed with the Tatritin peptide diluted serially to 1, 2, 4, 8, and 16 μM in 20 mM Tris–HCl. After incubation at 37°C for 2 h, the bacterial suspension was spread on Sabouraud glucose agar with chloramphenicol (4% dextrose, 1% pancreatic digest of casein, 1.5% agar, and 0.1% chloramphenicol) and incubated at 37°C for 24 h.

To explore the killing kinetics of Tatritin, bacteria were prepared as described above. Then 20 μl of bacteria (5 × 10^6^ CFU/ml) was mixed with 80 μl of the peptide at the concentration of 20 μM and incubated at 37°C. At the indicated time, bacteria were serially diluted and placed on Mueller–Hinton broth agar plates for the viability measurement.

### Membrane Permeabilization Assay

To analyze the effect of Tatritin on the permeability of bacterial membranes, flow cytometry was performed as previously described (38, 39). Briefly, bacteria were cultured to OD_600_ of 0.6–0.8, centrifuged, washed, and resuspended in PBS. Approximately 5 × 10^7^ cells in 50 µl PBS was added to an EP tube containing 150 µl of Tatritin (10 µM for the final concentration). Bacterial suspension without peptides was used as a control. After incubation at 37°C for 30 min, propidium iodide (PI; Beyotime, China) was added to a final concentration of 10 μg/ml. The influx of PI into bacterial cells was investigated by a CytoFLEX S flow cytometer (Beckman Coulter, Brea, CA, USA) at 10,000 events. The cell penetrating efficiency was analyzed by the FlowJo software package (Tree Star).

A scanning electronic microscope (SEM) was used to observe the microscopic structure and morphological changes of bacterial cells after Tatritin treatment. Approximately 5 × 10^5^ CFU of cells was incubated with 20 µM Tatritin (50 mM Tris–HCl, pH 7.2) for 2 h at 37°C. Subsequently, the bacterial cells were centrifuged and fixed with 2.5% glutaraldehyde overnight at 4°C, dehydrated, vacuum dried, sputter-coated with gold, and then observed with an S-4800 field-emission SEM (Hitachi, Tokyo, Japan) at an accelerating voltage of 20 kV.

### Localization of Tatritin Peptide on Bacteria

The peptide localization on bacteria was monitored by a confocal fluorescence microscope as previously described (40). Approximately 1–5 × 10^8^ CFU (*E. coli* and *S. aureus*) was resuspended in PBS containing FITC-labeled Tatritin at a concentration of 2.5 µM. Following incubation for 1 h at 37°C, cells were washed, fixed, and immobilized on poly-L-lysine-coated glass slides. After staining with DAPI (10 µg/ml), antifade mounting medium (Beyotime, China) was added to the slides prior to covering. Localization of labeled-peptide FITC-Tatritin was observed using an N-STORM confocal microscope (Nikon, Melville, NY, USA).

### LPS-Binding Activity

The LPS-binding activity of Tatritin was measured through ELISA using rabbit anti-Tatritin antibody and HRP-conjugated goat anti-rabbit IgG as previously described (41). Binding of peptides to LPS was expressed as a percentage of absorbance developed by 0.2 µg peptide. All reactions were performed in triplicate.

### DNA-Binding Assay

Gel retardation experiments were performed to determine the binding of the Tatritin peptide with DNA. The genomic DNAs of *E. coli* and *S. aureus* (200 ng) were mixed respectively with increased amounts of peptides in 20 μl binding buffer (10 mM Tris–HCl, 1 mM EDTA, 1 mM DTT, 20 mM KCl, 50 µg/ml BSA, 5% glycerol, pH 8.0) for 1 h at 37°C. The migration of DNA was assessed by 1% agarose gel electrophoresis.

### Hemolysis and Cytotoxicity Assays

The hemolytic activity of Tatritin was determined using erythrocytes of grass carp (*Ctenopharyngodon idella*), blunt snout bream (*Megalobrama amblycephala*), and domestic pig (*Sus scrofa*), as previously described (38, 42). The absorbance was measured at 405 nm using an Infinite F200 microplate reader (Tecan, Austria). Percentage hemolysis of Tatritin was determined by measuring the OD_405_ ratio of the supernatant. The negative and positive controls were incubated with PBS and 2% Triton X-100, respectively.

Cell lines including porcine kidney cells (PK15, *S. scrofa*), fathead minnow cells (FHM, *Pimephales promelas*), grass carp liver cells (L8824, *C. idella*), zebrafish embryonic fibroblast cells (ZF4, *Danio rerio*), and zebrafish liver cells (ZFL) were used to assess the cytotoxicity of Tatritin. A cell suspension (1 × 10^5^ cells/ml) was added to each well of a 96-well culture plate (100 µl per well) and incubated overnight. Then, a serial dilution of Tatritin in PBS was added to each well with peptides at the final concentrations of 1, 2, 4, 8, 16, 32, and 64 µM. Cells with Tatritin were cultured for 48 h under the conditions of 5% CO_2_ at 28°C except PK15 for 37°C. Then, cell viabilities were assessed using a CCK-8 kit (Biosharp, China) according to the manufacturer’s instructions. Finally, OD_490_ was measured using an Infinite F200 microplate reader (Tecan, Grödig, Austria). Controls were included as described in hemolysis assays. The relative cell viability was determined by comparison with 0% and 100% lysis using the following formula: [(A_Tatritin_ – A_100% lysis_)/(A_0% lysis_ – A_100% lysis_)] × 100, where A is the absorbance at 490 nm.

### Therapeutic and Preventive Effects of Tatritin in Zebrafish Infected With *A. hydrophila*

Zebrafish (0.4 ± 0.05 g) were bred and maintained under a 14:10-h light/dark cycle at 24°C. All experiments were performed following the recommendations in the Guide for the Care and Use of Laboratory Animals of the National Institutes of Health.

In order to evaluate the therapeutic effects of Tatritin, zebrafish (n = 20) were injected with 5.6 × 10^4^ CFU of *A. hydrophila*, followed by the administration of Tatritin (0.2 µg/g), or equal volumes of PBS (control) at 3 h postinfection by intraperitoneal injection (43). The dilution-coated plate method was used to quantify bacterial load in tissues as previously described (40). Briefly, liver and gut tissues of zebrafish were weighed and homogenized in PBS. The tissue homogenate was serially diluted and plated onto LB agar plates for obtaining colony counts.

Similar to the therapeutic effect experiment of Tatritin, a preventive experiment was performed *via* infection with *A. hydrophila* at 24 h post-peptide injection, and the bacterial load in the tissues was detected.

### Statistical Analysis

Data were presented as the means ± SD. The statistical significance was assessed by two-tailed independent t-test. *p* < 0.05 value was considered as statistically significant difference.

## Results

### Sequence and Structure Analysis of Tatritin

**Figure 1:**
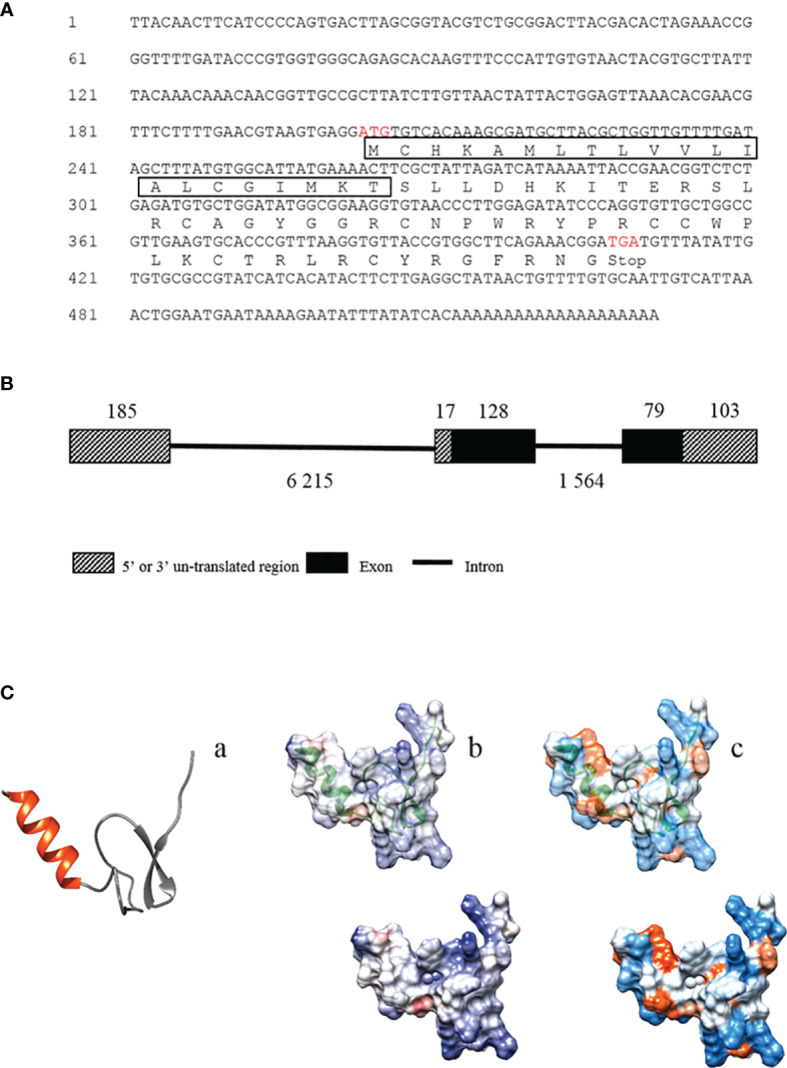
The mRNA and deduced amino acid sequences of *Tatritin* and the structural analysis. **(A)** The mRNA and deduced amino acid sequences of *Tatritin*. The box shows the predicted signal peptide, and the red letters show the predicted start and stop codons. Schematic diagram of the gene **(B)** and peptide structure **(C)** of Tatritin. Tatritin with *α*-helix in red and cys-stabilized *β*-sheet in gray (**C**-a); analysis of electrostatic surface, positive charges in blue and negative charges in red (**C**-b); hydrophobicity surface analysis, hydrophobic regions with warmer colors (**C**-c).

In the previous study, we performed a multi-omics (transcriptome and peptidome) analysis of Chinese horseshoe crab hemolymph to identify genes induced by bacterial infection and found that the gene, termed *Tatritin* in this study, was significantly (*p* < 0.05) induced by pathogenic *V. parahaemolyticus* (29). The open reading frame (ORF) and 3′ UTR sequences of *Tatritin* (accession number OM128176) were identified *via* RT-PCR. The primers using in this study were showed in **Table 1**. In addition, the genomic DNA sequence was obtained from the Chinese horseshoe crab genome (1) as an unannotated sequence. The results showed that the length of *Tatritin* mRNA was 531 bp with ORF of 207 bp encoding 68 amino acids (aa) (**Figure 1A**). The preprotein of Tatritin consists of a signal peptide (21 aa) and a mature peptide (47 aa) enriched by cysteines. Although the *Tatritin* gene consists of 3 exons, the preprotein is encoded by only the second and third exons (**Figure 1B**). Moreover, the putative mature peptide was 5.6 kDa with a theoretical isoelectric point (pI) of 9.99. The mature peptide sequence of Tatritin showed a *α*-helix structure in the N-terminal and an anti-parallel *β*-sheet structure in the cys-stabilized C-terminal region (**Figure 1C** **-**a). In addition, the views of surface of the coulombic electrostatic potential (**Figure 1C** **-**b) and amino acid hydrophobicity (**Figure 1C** **-**c) showed that most positive-charge and almost as many hydrophilic and hydrophobic amino acids were exposed to the outside of the protein. This conformation suggests an interaction with the bacterial membrane (33). These characteristics of cys-stabilized, small size, cationicity, and amphipathic structure were considered to be the basic conditions of antimicrobial peptides (44).

**Table 1:** Primers used for amplification of *Tatritin* and qRT-PCR in this study.

Names	Sequences (5′~3′)	Description	Tm (˚C)
Tatritin_ORF	F: CCAGTGACTTAGCGGTACG R: AGCCTCAAGAAGTATGTGATGA	Amplification of *Tatritin* ORF	58~60
Tatritin_3’end	F1: GGCAGAGCACAAGTTTCC F2: GCGATGCTTACGCTGGTTGTTTTGA	3′ end specific upstream primers	56~58
Tatritin_qRT-PCR	F: GTTACACCTTCCGCCATAT R: GTTGCCGCTTATCTTGTTAA	Expression analysis of *Tatritin*	58~62
*Gapdh*	F: ATCATCAGCAATGCCTCTTG R: GCCTTAGAGCTTGGTCCATC	Reference gene	58~62

### Tissue Distribution and Expression Pattern of Tatritin

**Figure 2:**
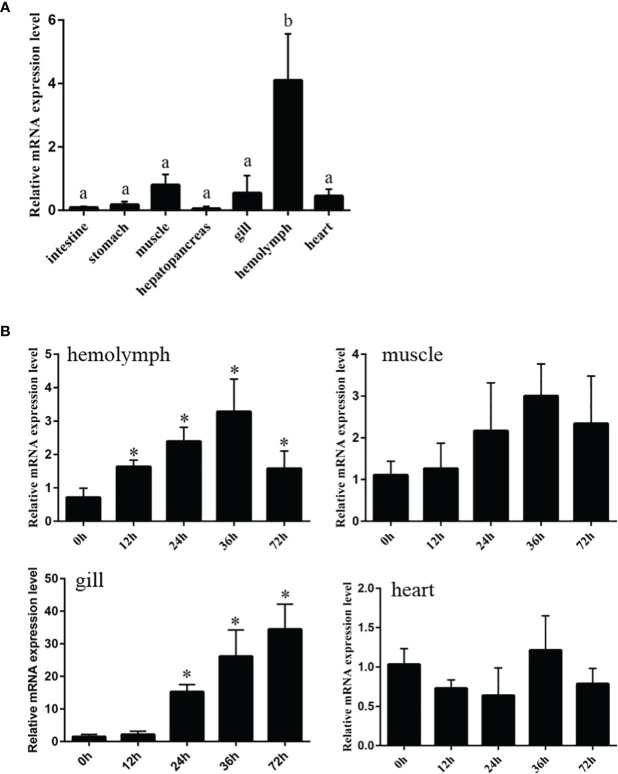
Expression patterns of the *Tatritin* gene in healthy and infected Chinese horseshoe crab with *A. hydrophila*. **(A)** The expression of *Tatritin* was measured by qRT-PCR in seven tissues, intestine, stomach, muscle, hepatopancreas, gill, hemolymph, and heart from healthy horseshoe crabs. **(B)** The expression pattern of *Tatritin* in hemolymph, gill, muscle, and heart infected with *A. hydrophila*. *Gapdh* was used as the internal reference. Each experiment was executed in triplicate. Data are shown as mean ± SD (N = 3). The asterisk and different letters indicate significant difference (*p < 0.05) compared with the control (set as 1).

The mRNA expression of *Tatritin* in a range of Chinese horseshoe crab tissues was analyzed by qRT-PCR. *Tatritin* mRNA was mainly expressed in hemolymph, muscle, gill, and heart and slightly expressed in stomach, hepatopancreas, and intestines (**Figure 2A**). After infecting with *V. parahaemolyticus*, the mRNA expression levels of *Tatritin* were increased significantly (*p <*0.05) in hemolymph (all time points postinfection) and gills (24, 36, and 72 h postinfection) (**Figure 2B**). However, there is no significant difference in the mRNA expression levels of *Tatritin* in muscle and heart.

### Immunofluorescence Localization of Tatritin in Muscle and Gill

**Figure 3:**
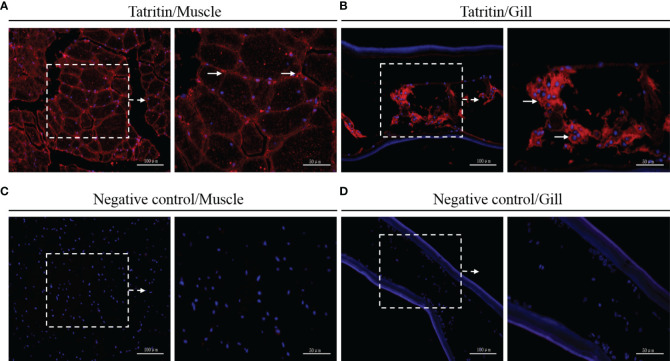
Immunofluorescence detection of Tatritin in muscle and gill. Cryosections were stained with specific antibody for Tatritin (red) and with DAPI for nuclei (blue). Signals of Tatritin peptide were observed in muscle **(A)** and gill **(B)**. Controls were stained with anti-GST antibody in muscle **(C)** and gill **(D)**. The right image of each panel shows the enlarged image of the area outlined in the left image. The long arrows indicate location expressing Tatritin in endomysium and sarcoplasm of muscle or in the cavity of gill.

The expression and localization of Tatritin in muscle and gill tissues of Chinese horseshoe crab were further analyzed by immunofluorescence assay. In muscle, the immunoreactive signal of Tatritin was observed in the endomysium and sarcoplasm (**Figure 3A**). In gill, the positive signal was only found in the cavity (**Figure 3B**). Moreover, the negative control showed no visible immunoreactive signal in muscle or gill (**Figures 3C, D** **)**.

### Antimicrobial Activities

**Figure 4:**
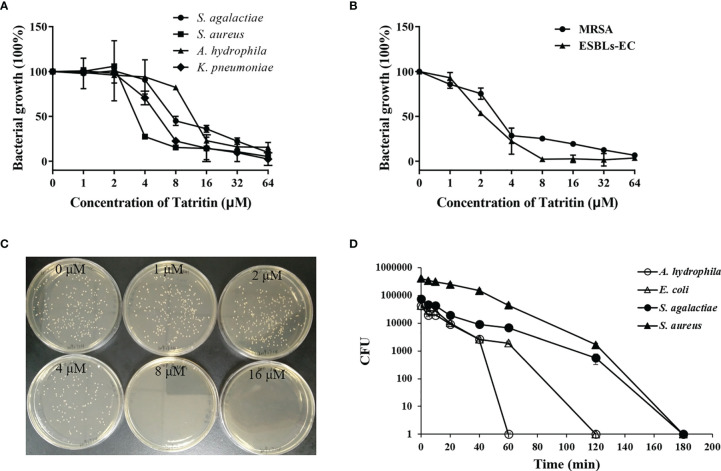
Antimicrobial activities of Tatritin against *S. aureus*, *S. agalactiae*, *A. hydrophila*, *K. pneumoniae*, ESBLs-EC, MRSA, and *C. albicans*. Antibacterial activity of Tatritin peptide against Gram-positive, -negative bacteria **(A)** and drug-resistant bacteria **(B)**. The bacteria in the mid-logarithmic phase were diluted in medium and incubated with serially diluted peptide at a concentration of 10^5^ CFU/ml for 18 h. OD_595_ was measured and corrected by initial OD_595_ values. Bacterial growth rates were calculated as the bacterial density in the presence of peptide to the bacterial density of controls. Data shown are the means of duplicate reactions in a representative of three independent experiments. **(C)** Antifungal activity of Tatritin peptide. Approximately 2 × 10^5^ cells of *C. albicans* were mixed with Tatritin peptide diluted serially to 1, 2, 4, 8, and 16 μM. The minimum concentration in petri dish without colonies was regarded as the minimum fungicidal concentration. **(D)** The killing kinetics of Tatritin against Gram-positive and -negative bacteria. Bacteria were treated with Tatritin peptide (20 μM). The viability of bacteria was measured at indicated time points. Samples were measured in triplicates.

The antimicrobial assay showed that the Tatritin peptide was antibacterial to all strains tested (**Figure 4A**), including Gram-positive bacteria (*S. aureus* and *S. agalactiae*) and Gram-negative bacteria (*A. hydrophila*, *K. pneumoniae*). Similarly, the growth of drug-resistant strains including ESBLs-EC and MRSA was also inhibited markedly by the Tatritin peptide (**Figure 4B**). The 50% minimum inhibitory concentrations (MIC_50_) for all strains tested were 4–16 μM. In addition, *C. albicans*, a stubborn pathogenic fungi, can also be killed by Tatritin peptide at a minimum concentration of 8 μM (**Figure 4C**).

The time course of bacterial viability was determined after these bacteria (*A. hydrophila*, *E. coli*, *S. aureus*, and *S. agalactiae*) were treated with the Tatritin peptide (**Figure 4D**). The results showed that *A. hydrophila* was immediately killed within 60 min upon the addition of Tatritin peptide. Although *E. coli* belonged to Gram-negative bacteria, it was killed by Tatritin peptide in 120 min. For *S. aureus* and *S. agalactiae*, complete killing by Tatritin peptide was observed in 180 min.

### Hemolytic and Cytotoxic Activities of Tatritin

**Figure 5:**
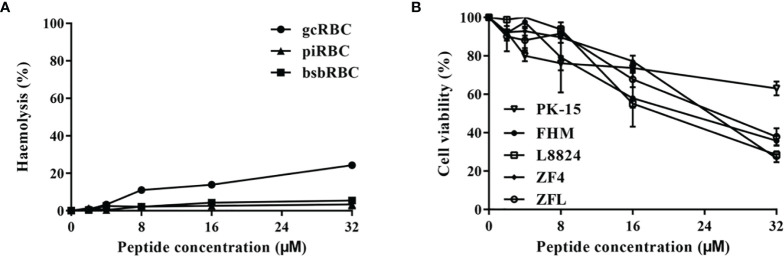
The hemolytic and cytotoxic activities of Tatritin against erythrocytes and cell lines from fish and pig. **(A)** The Tatritin peptide was added to the erythrocytes of grass carp (gcRBC), blunt snout bream (bsbRBC), and domestic pig (piRBC) and incubated at 24˚C for 2 h, and then OD_405_ of the supernatant was measured. **(B)** The Tatritin peptide was added to the cells of PK15 (*Sus scrofa*), FHM (*Pimephales promelas*), L8824 (*Ctenopharyngodon idella*), ZFL (*Danio rerio*), and ZF4 (*D. rerio*) and incubated at 28˚C for 48 h; CCK-8 solution was then added and incubated at 28˚C for an additional 4 h. The absorbance was subsequently measured at 450 nm. Data shown are the means ± SD (n = 3).

As shown in **Figure 5A**, Tatritin peptide did not exhibit apparent hemolytic activity against erythrocytes from three species tested at a low concentration (<32 μM). However, cytotoxicity was observed at 8–32 µM of concentrations in all cell lines tested, although the cytotoxicity of Tatritin was slight at the concentration <8 µM (**Figure 5B**). Moreover, the cytotoxicity of Tatritin to cell lines from fish (including FHM, L8824, ZF4, and ZFL) was more pronounced than PK15 from pig (**Figure 5B**).

### Localization and Mechanism of Tatritin in Antimicrobial Process

**Figure 6:**
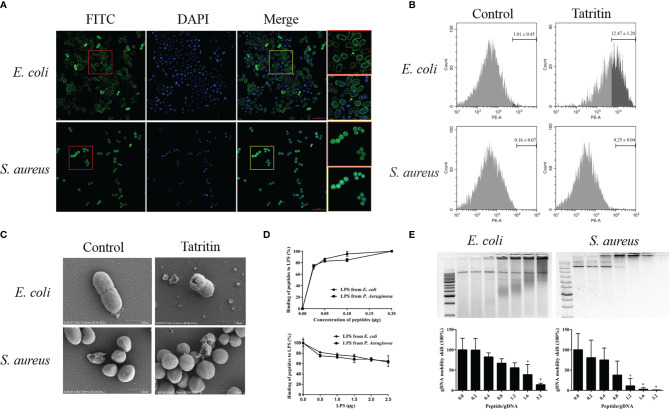
Function and mechanism of Tatritin in antibacterial process. **(A)** Localization of Tatritin peptide on the bacteria. Approximate 1–5 × 10^8^ CFU of *E. coli* ATCC35218, and *S. aureus* ATCC6538 were incubated with FITC-labeled Tatritin (2.5 μM) for 1 h. The bacteria were washed, fixed, and stained with DAPI (blue). Images were taken using confocal microscope. The cells in the red and yellow boxes were enlarged. Scale bar is 5 μm. **(B)** PI labeling of bacterial cells treated with Tatritin peptide. Approximately 5 × 10^7^ cells in 50 µl PBS were added to an EP tube containing 150 µl of Tatritin (10 µM). Then PI was added, and the influx of PI into bacterial cells was investigated by flow cytometry. All bacteria were gated, and the cell-penetrating efficiency was determined using the data from three tests. **(C)** Scanning electron microscope of *E. coli* and *S. aureus* cultured with or without Tatritin (20 µM) for 2 h at 37˚C. **(D)** Evaluation of the LPS-binding activity of Tatritin. The LPS-binding activities of Tatritin were investigated by incubating 0.05–0.2 µg of Tatritin in the LPS-immobilized 96-well microtiter plates (0.2 µg LPS/well) for 1 h at 37°C in 50 µl of RPMI 1640. The bound peptides were detected by TMB reaction using rabbit anti-Tatritin and HRP-conjugated goat anti-rabbit IgG. Tatritin (0.2 µg/well) was incubated in the LPS-immobilized 96-well microtiter plates for 1 h at 37°C in the absence or the presence of LPS (0.5–2.5 µg/well) in 50 µl of RPMI 1640, and the bound peptide was detected as described above. Binding of Tatritin to the LPS-immobilized plates was expressed as a percentage of that incubated with 0.2 µg of each peptide in the absence of added LPS. Data are the mean ± SD (n = 3). **(E)** Gel shift assay of bacterial gDNA of *E. coli* and *S. aureus* mixed with increasing concentrations of Tatritin. The abscissa represents the peptide/gDNA ratio. ImageJ was used to analyze the intensity of nucleic acid bands in gel shift assay. The abscissa represents the peptide/gDNA ratio and the ordinate represents the gDNA mobility shift, *P<0.05.

*E. coli* and *S. aureus* were treated with FITC-labeled Tatritin, and the localization of Tatritin on bacteria was visualized using a confocal fluorescence microscope. The results (**Figure 6A**) showed that, after the peptide treatment, *E. coli* appeared as hollow rods with fluorescence clearly defined bacteria surface, suggesting that Tatritin was accumulated on the membrane. Interestingly, unlike *E. coli*, the Tatritin peptide was localized inside the cells of *S. aureus*.

To evaluate the penetrating efficiencies of Tatritin to bacteria (*E. coli* and *S. aureus*), the relative fluorescence intensities were detected by flow cytometry. The results indicated that the membrane penetrating efficiency of Tatritin for *E. coli* was significantly higher than *S. aureus* (**Figure 6B**).

The morphology of two bacteria treated with Tatritin was imaged by SEM. The results showed that Tatritin treatment led to obvious changes of the cell surface characteristics in *E. coli*. Cell morphology disruption and cell wall collapse were observed (**Figure 6C**). Interestingly, there was no obvious morphology change in *S. aureus* (**Figure 6C**). These data suggest that Tatritin peptide may have different antibacterial mechanisms against Gram-negative and -positive bacteria.

To clarify the mechanism of Tatritin binding to bacteria, the LPS-binding activity of Tatritin peptide was investigated using LPS-immobilized microtiter plates. The results showed that Tatritin bound to the LPS-immobilized plates in a dose-dependent fashion (**Figure 6D**). Interestingly, when the peptide concentration was constant, the binding activity gradually decreased as the LPS concentration increased (**Figure 6D**). It was suggested that the bacteria can be inhibited by Tatritin *via* directly binding LPS in the cell wall. In addition, the binding ability of Tatritin can be inhibited by excessive LPS.

On the other hand, the DNA-binding activity of Tatritin was also detected *via* gel retarding assay. The mobility of DNA from two bacteria was decreased when the ratio of peptide/DNA increased, although the mobility of two bacteria DNA had obvious difference (**Figure 6E**). The *S. aureus* DNA was completely retarded at the ratio of 1.2, suggesting that Tatritin has a strong binding activity to *S. aureus* DNA (**Figure 6E**).

### Tatritin Is a Prophylactic and Therapeutic Agent Against *A. hydrophila* Infection in Zebrafish

To investigate the protective and therapeutic effects of the Tatritin peptide *in vivo*, an infection model of *A. hydrophila* in zebrafish (**Supplementary Figure S1**) was established.

**Figure 7:**
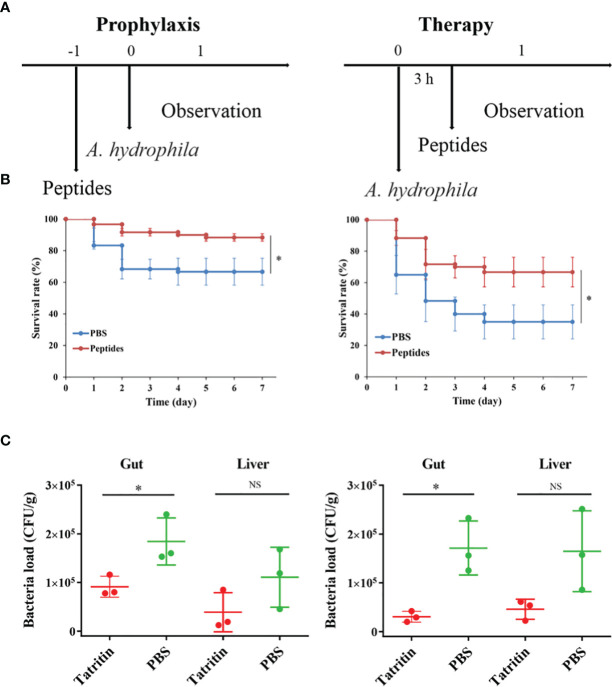
Protective efficacy of Tatritin in the zebrafish model of infection. **(A)** Schematic of the *A*. *hydrophila*-infected prophylactic and therapeutic model. **(B)** Preventive efficacy of Tatritin in the *A. hydrophila*-infected prophylactic and therapeutic model. Zebrafish were received intraperitoneal injection at a single dose of 2 µg/g Tatritin or equal volumes of PBS (n = 20) at 24 h before or 3 h after the pathogenic bacteria of *A. hydrophila* was injected with a lethal dose of 5.6 × 10^4^ CFU. The mortality in each group was recorded for at least 7 days. Experiments were conducted in triplicates, and the data shown are the means. Asterisk indicates that the difference is significant. **(C)** At 3 days postinfection, bacterial loads in gut and liver of zebrafish infected with *A. hydrophila* in prophylaxis and therapy groups were detected, (n = 3). *P<0.05, NS, not significant.

After being infected with *A. hydrophila*, the fish showed ulcer on skin, ascites, intestinal bleeding, and bacterial overload in the digestive organs and eventually died within a short period of time (45). In the prevention test, Tatritin peptide was injected intraperitoneally into zebrafish at 24 h before infection with *A. hydrophila* (**Figure 7A**). The survival rate of zebrafish treated with Tatritin prior to bacterial inoculation (preventive effects) was 88.3% in 7 days (**Figure 7B**) and significantly increased compared with the control group (*p* < 0.05). Similarly, in the therapeutic test, the survival rate of the Tatritin group was significantly higher than that of the control group (*p* < 0.05), and the survival rates were 66.7% and 35% (**Figure 7B**), respectively.

In addition, to examine the effects of Tatritin in reducing the virulence of *A. hydrophila* on the zebrafish, the bacterial loads in organs were evaluated after infection. The results showed that the bacterial loads in the gut and liver from Tatritin groups were reduced compared with the control groups, although the difference was not significant in liver tissue (**Figure 7C**).

## Discussion

The antimicrobial peptide named Tatritin was newly identified from the transcriptome of Chinese horseshoe crab (29) and showed a significantly upregulated expression (*p* < 0.05) in hemolymph of Chinese horseshoe crab after infection with Gram-negative bacteria. The *Tatritin* gene consists of three exons, coding 68 amino acid residues. Tatritin contains a signal peptide of 21 aa in the N-terminal and a cys-stabilized mature peptide of 47 aa in the C-terminal. The signal peptide is essential to guiding its targeting transport after AMP synthesis (46, 47). The physical and chemical properties of Tatritin mature peptide, including small size (5.6 kDa), cys stability, high isoelectric point (9.99), the special structure of *α*-helix, *β*-sheet, and amphipathicity, suggested that it may be a new antimicrobial peptide (44). Although many antimicrobial peptides from Chinese horseshoe crab (including Tachyplesins, Tachycitin and Tachystatins) have been reported (16, 25, 26), Tatritin has relatively low similarity (less than 35%) to other sequences based on sequence alignments.

Marine invertebrates rely solely on the innate immune system that includes both humoral and cellular responses (4). As the first barrier of the humoral immune system of invertebrates, antimicrobial peptides can be quickly activated to eliminate pathogens from hosts. After infecting with Gram-negative bacteria, Tatritin was highly expressed in hemolymph, muscle, and gill, indicating that it plays an important role in the defense system of Chinese horseshoe crab against pathogenic microbes. As known, gill is the main tissue of oxygen exchange in Chinese horseshoe crab, and the soft carapace and dense vessels in the gills are one of the main places for pathogens to invade. Therefore, the AMPs may play an important role in gills as the first-line defense against pathogens. Similar to the cathelicidin antimicrobial peptides in muscle of rainbow trout (*Oncorhynchus mykiss*) (38), Tatritin was highly expressed in muscle of Chinese horseshoe crabs. The gills and muscle have a direct fluid exchange with hemolymph, which indicated that Tatritin may participate in the daily defense process as the important immune factor. The antimicrobial activity results revealed that Tatritin peptide had a strong antimicrobial activity against not only Gram-negative and -positive bacteria but also fungi, and even drug-resistant bacteria, including ESBLs-EC and MRSA, can be killed by Tatritin as effective as antibiotic-sensitive strains. Although Tatritin peptide has similar inhibitory effects on Gram-negative and -positive bacteria, it showed greater sensitivity to Gram-negative bacteria. It has been previously shown that AMPs causing membrane permeabilization exhibited fast killing kinetics, while AMPs targeting intracellular components exhibited slow killing kinetics (26). In the present study, Tatritin peptide can directly bind to Gram-negative bacteria *via* recognizing LPS and killing the bacteria by destroying their cell membranes. The similar antibacterial mechanism of AMPs has been reported in other studies (38, 48, 49). However, unlike Gram-negative bacteria, Gram-positive bacteria were more likely to be killed by Tatritin through the combination of Tatritin and gDNA. Similarly, many antimicrobial peptides, including pleurocidin, NK-18, tachyplesins, and piscidin, have been found to kill bacteria by binding with DNA (50–53). Although interaction with nucleic acids is the main antibacterial mechanism, these AMPs can still cause membrane damage and/or changes in membrane potential to a certain extent (50–54). Interestingly, *S. aureus* treated with Tatritin did not show obvious membrane damage. Similarly, the AMP mBjAMP1 from *Branchiostoma japonicum* showed effective growth inhibition and bactericidal activities against pathogenic bacteria but did not disrupt membrane integrity (55). The *α*-hairpinin-like scaffold stabilized by an intramolecular disulfide bond in mBjAMP1 was considered to be the main reason for passing through the cell membrane without membrane damage. However, the speculative hairpin structure of Tatritin is formed by three cys-stabilized disulfide bridges.

**Figure 8:**
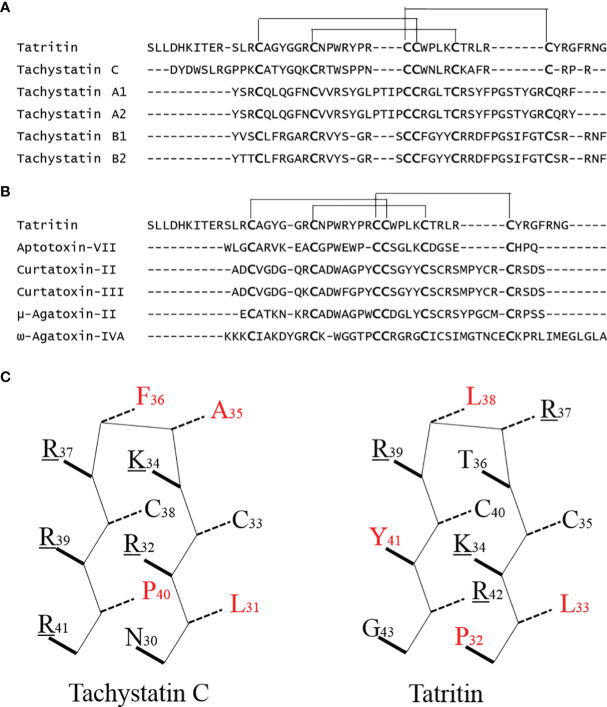
Sequence comparison of Tatritin, Tachystatins, and Neurotoxins from spider venoms. **(A)** Amino acid sequence alignment of Tatritin and Tachystatins. Conserved cysteines are indicated in large boldface letters. Lines show the identical pattern of disulfide bonds in Tatritin and Tachystatins. **(B)** Alignment of the amino acid sequence of Tatritin with those of insecticidal peptides from venom of the spiders. Conserved cysteines are indicated in large boldface letters. Lines show the identical pattern of disulfide bonds in Tatritin and Neurotoxins. **(C)** *β*-Sheet structural models of the C-terminal regions of Tatritin and Tachystatin C. Solid/dashed lines indicate side chains pointing out of/into the plane of the diagram. Basic and hydrophobic amino acid residues are indicated in underline and red highlight, respectively. The numbers indicate the positions of amino acid residues in the mature peptide.

Although no antibacterial peptide with high similarity with Tatritin has been reported in the previous study, the conservative cys-stabilized motif (inhibitory cystine knot, ICK) of Tachystatin C from horseshoe crab was almost the same as Tatritin (**Figure 8A**). Besides, the other two Tachystatins in horseshoe crab, Tachystatin A and Tachystatin B, and some neurotoxins, such as ω-Agatoxin-IVA, μ-Agatoxin-II, Aptotoxin-VII, and Curtatoxin (**Figure 8B**), from spider venom also have the same motif of cysteines (26). The horseshoe crab has a close relationship with spiders in evolutionary history (Chelicerata); thus, these peptides such as Tatritin, Tachystatins, and spider toxin are considered to have evolved from the common ancestor of ancient arthropods as antimicrobial substances. The same region or motif is likely to form three disulfide bonds in Tatritin (**Figure 8A**) and cause Tatritin to have similar functions, such as antimicrobial activity, hemolytic activity, and the binding activity of LPS or chitin, to Tachystatins. As predicted, the antimicrobial activity, LPS-binding activity, and slight hemolytic activity of Tatritin were observed similar to Tachystatin C in low peptide concentrations. On the other hand, the cytotoxic activity of Tatritin was more serious than hemolytic activity in the same peptide concentrations. In addition, compared with fish cells, Tatritin had less hemolytic and cytotoxic activities to mammalian cells. The sequence feature of a cationic site flanked by a hydrophobic surface was considered to be the main reason for the existence of the cytolytic property for antibacterial peptide (26). Among all three Tachystatins, only the amphiphilic *β*-sheet in the C-terminal of Tachystatin C may be the structure basic for cytolytic activity (26, 56). Interestingly, although the Tatritin peptide has hemolytic and cytotoxic activities like Tachystatin, it does not have this special amphiphilic structure at the C-terminal (**Figure 8C**). In previous studies, peptides containing the short antiparallel *β*-sheet with the ICK motif of the Tachystatin family and the toxin of ω-Agatoxin IVA can directly bind with chitin and display antifungal activity (57, 58). Based on the high similarity of the special structure and the antifungal activity of Tatritin in this study, we conjectured that Tatritin can also directly combine with chitin, although the mechanism of chitin binding has not been clarified. Sequence alignment with the Tachystatin family and ω-Agatoxin IVA suggested that the residues of Trp^24^ and Tyr^26^ in Tatritin might be important for the chitin-binding property (55). Chitin is a component of the cell wall of fungi, and it is also the major structural component of arthropod exoskeletons. Tatritin peptide expressed in many tissues may play a role not only in against invading pathogen but also in injury repair like Tachystatins (26).

In order to apply the AMP in the field of aquaculture, we further verified the protective and therapeutic effects of Tatritin on zebrafish infected with a lethal dose of *A. hydrophila*. As expected, prophylactic or therapeutic administration of Tatritin increased the survival rate of zebrafish with a lower bacterial cell number in the gut and liver. The therapeutic effect of Tatritin is better than its protective effect, although Tatritin peptide can effectively improve the survival rate of zebrafish infected with *A. hydrophila* in two groups. Moreover, the effective protection rates (survival rate difference between the peptide and PBS groups) of two groups injected with Tatritin peptide compared with PBS groups were 21.6% and 31.7%, respectively. Similarly, the antimicrobial peptide hepcidin from *C. idella* can significantly reduce the mortality of fish infected with bacteria, and its therapeutic effect is also significantly better than its protective effect (43). These results demonstrate that the antimicrobial peptides have an effective defense effect against pathogen invasion, which can improve the survival rate of bacteria-infected fish and increase the efficiency of pathogen eliminating.

In conclusion, the novel antimicrobial peptide of Tatritin was identified and characterized in *Tachypleus tridentatus*. Tatritin was significantly induced after bacterial infection in hemolymph and gill. Tatritin exhibited a broad spectrum of antimicrobial activity against Gram-negative and -positive bacteria and fungi. Furthermore, Tatritin may recognize and inhibit pathogenic microorganisms by directly binding to LPS, DNA, and chitin. In addition, the Tatritin peptide can significantly reduce the bacteria load in tissues and increase the survival rate of zebrafish after bacterial infection. Due to its broad-spectrum antimicrobial activity *in vivo* and *in vitro* and the sensitivity to drug-resistant strains, the Tatritin peptide can be used as a new type of drug for infection treatment or as an immune enhancer in animals like fish. The excessive use of antibiotics may also be improved if AMPs, like Tatritin, are used in aquaculture.

## Data Availability Statement

The datasets presented in this study can be found in online repositories. The names of the repository/repositories and accession number(s) can be found in the article/**Supplementary Material**.

## Ethics Statement

The Huazhong Agricultural University Sciences Animal Care Committee provided official ethics board approval for this study.

## Author Contributions

All authors have read and approved the manuscript. W-FW designed the experiments, analyzed the experiments data, and wrote the manuscript. X-YX, YH, Y-KL, HL, and X-LC performed, analyzed, and interpreted the study. H-LW designed the experiments and revised the manuscript. All authors contributed to the article and approved the submitted version.

## Funding

This work was supported by the Science and Technology Major Project of Guangxi (No. AA17204088) and Fundamental Research Funds for the Central Universities (2662019PY036).

## Conflict of Interest

The authors declare that the research was conducted in the absence of any commercial or financial relationships that could be construed as a potential conflict of interest.

## Publisher’s Note

All claims expressed in this article are solely those of the authors and do not necessarily represent those of their affiliated organizations, or those of the publisher, the editors and the reviewers. Any product that may be evaluated in this article, or claim that may be made by its manufacturer, is not guaranteed or endorsed by the publisher.

## Supplementary Material

Supplementary Figure 1 The model of *A. hydrophila* infection test in zebrafish. Zebrafish infected with *A. hydrophila* showed ulcer on skin, accumulation of ascites, the intestinal bleeding, the bacterial overload in the digestive organs, and eventually died.

## Abbreviations

*T. tridentatus*, *Tachypleus tridentatus*; AMPs, antimicrobial peptides; gDNA, genomic DNA; LPS, lipopolysaccharides; *V. parahaemolyticus*, *Vibrio parahaemolyticus*; qRT-PCR, quantitative real-time polymerase chain reaction; SD, standard deviation; *A. hydrophila*, *Aeromonas hydrophila*; *E. coli*, *Escherichia coli*; *K. pneumoniae*, *Klebsiella pneumoniae*; *S. aureus*, *Staphylococcus aureus*; *S. agalactiae*, *Streptococcus agalactiae*; *C. albicans*, *Candida albicans*; pI, propidium iodide; SEM, scanning electronic microscopy; *C. idella*, *Ctenopharyngodon idella*; *M. amblycephala*, *Megalobrama amblycephala*; MOI, multiplicity of infection; ORF, open reading frame; *V. parahaemolyticus*, *Vibrio parahaemolyticus*; ICK, inhibitory cystine knot; MIC50, 50% minimum inhibitory concentration; MRSA, methicillin-resistant *Staphylococcus aureus*; ESBLs-EC, extended spectrum *β*-lactamases.
