# Supplementary figures and images for: Identification of a Novel Antimicrobial Peptide From the Ancient Marine Arthropod Chinese Horseshoe Crab, Tachypleus tridentatus
Source: Front Immunol. 2022 Mar 23;13:794779. doi: 10.3389/fimmu.2022.794779 (PMC8984021; doi:10.3389/fimmu.2022.794779)

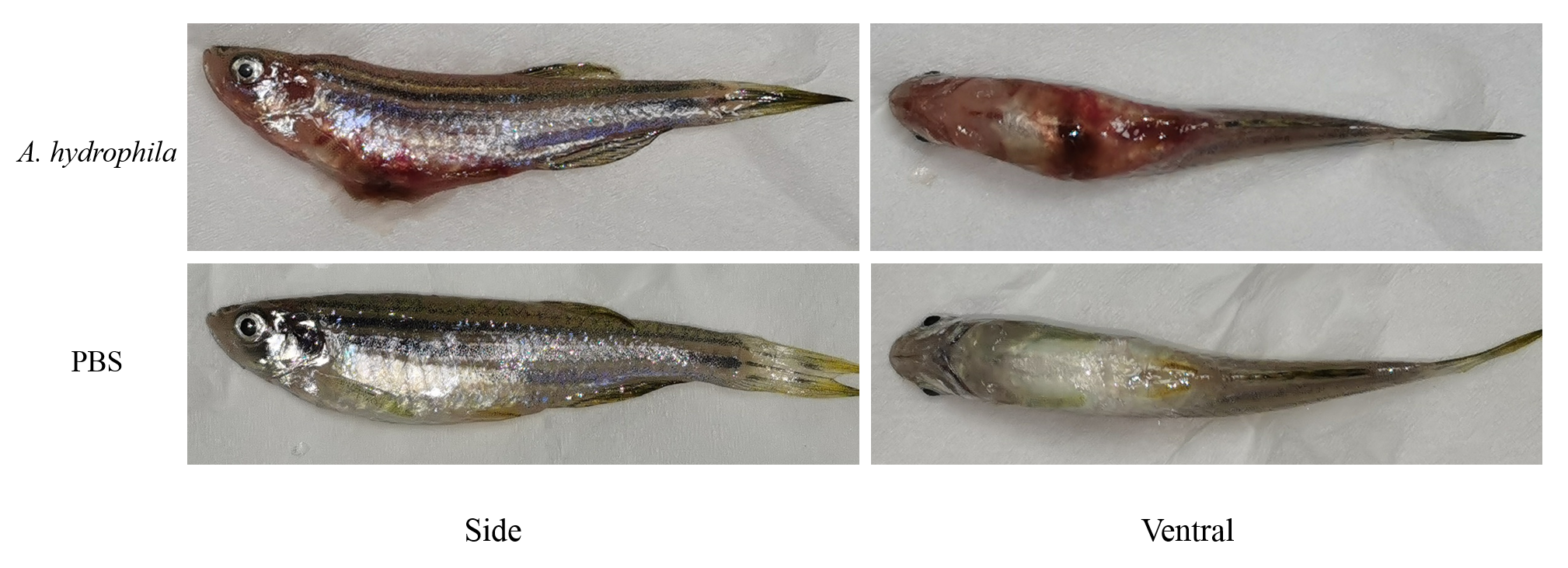

Supplement: Supplementary Figure 1 — The model of A. hydrophila infection test in zebrafish. Zebrafish infected with A. hydrophila showed ulcer on skin, accumulation of ascites, the intestinal bleeding, the bacterial overload in the digestive organs, and eventually died. [file Image_1.tif]
